# High resolution, 3-dimensional Ferumoxytol-enhanced cardiovascular magnetic resonance venography in central venous occlusion

**DOI:** 10.1186/s12968-019-0528-5

**Published:** 2019-03-11

**Authors:** Puja Shahrouki, John M. Moriarty, Sarah N. Khan, Biraj Bista, Stephen T. Kee, Brian G. DeRubertis, Takegawa Yoshida, Kim-Lien Nguyen, J. Paul Finn

**Affiliations:** 1 Diagnostic Cardiovascular Imaging Laboratory, University of California, Los Angeles, Peter V. Ueberroth Building Suite 3371, 10945 Le Conte Ave, Los Angeles, 90095-7206 CA USA; 2 Department of Radiological Sciences, University of California, Los Angeles, Los Angeles, USA; 3 Department of Medicine, University of California, Los Angeles, Los Angeles, USA; 4 Department of Surgery, University of California, Los Angeles, Los Angeles, USA; 5 David Geffen School of Medicine at UCLA, Los Angeles, USA; 6 VA Greater Los Angeles Healthcare System, Los Angeles, USA

**Keywords:** Central venous occlusion, Ferumoxytol, Chronic kidney disease, Magnetic resonance venography, Diagnostic accuracy

## Abstract

**Background:**

Although cardiovascular magnetic resonance venography (CMRV) is generally regarded as the technique of choice for imaging the central veins, conventional CMRV is not ideal. Gadolinium-based contrast agents (GBCA) are less suited to steady state venous imaging than to first pass arterial imaging and they may be contraindicated in patients with renal impairment where evaluation of venous anatomy is frequently required. We aim to evaluate the diagnostic performance of 3-dimensional (3D) ferumoxytol-enhanced CMRV (FE-CMRV) for suspected central venous occlusion in patients with renal failure and to assess its clinical impact on patient management.

**Methods:**

In this IRB-approved and HIPAA-compliant study, 52 consecutive adult patients (47 years, IQR 32–61; 29 male) with renal impairment and suspected venous occlusion underwent FE-CMRV, following infusion of ferumoxytol. Breath-held, high resolution, 3D steady state FE-CMRV was performed through the chest, abdomen and pelvis. Two blinded reviewers independently scored twenty-one named venous segments for quality and patency. Correlative catheter venography in 14 patients was used as the reference standard for diagnostic accuracy. Retrospective chart review was conducted to determine clinical impact of FE-CMRV. Interobserver agreement was determined using Gwet’s AC1 statistic.

**Results:**

All patients underwent technically successful FE-CMRV without any adverse events. 99.5% (1033/1038) of venous segments were of diagnostic quality (score ≥ 2/4) with very good interobserver agreement (AC1 = 0.91). Interobserver agreement for venous occlusion was also very good (AC1 = 0.93). The overall accuracy of FE-CMRV compared to catheter venography was perfect (100.0%). No additional imaging was required prior to a clinical management decision in any of the 52 patients. Twenty-four successful and uncomplicated venous interventions were carried out following pre-procedural vascular mapping with FE-CMRV.

**Conclusions:**

3D FE-CMRV is a practical, accurate and robust technique for high-resolution mapping of central thoracic, abdominal and pelvic veins and can be used to inform image-guided therapy. It may play a pivotal role in the care of patients in whom conventional contrast agents may be contraindicated or ineffective.

**Electronic supplementary material:**

The online version of this article (10.1186/s12968-019-0528-5) contains supplementary material, which is available to authorized users.

## Introduction

With expanding management options for patients with organ failure and malignancy, central venous occlusion is becoming an increasingly common and potentially devastating complication of treatment [1]. In patients with renal failure, imaging plays a crucial role in the assessment of central venous anatomy, but reliable visualization of the central veins is technically challenging and prone to failure. Whereas duplex ultrasonography is a first line test for peripheral veins, acoustic access in the chest and abdomen is often restricted, mandating the use of alternative imaging techniques [2, 3]. Computed tomography (CT) angiography is widely available and recent technical advances have greatly improved the speed and quality of arterial imaging, even with reduced doses of iodine based contrast media [4]. However, for CT venography, high contrast doses are still required, which in patients with kidney disease may further compromise renal function [4–7]. While non-contrast cardiovascular magnetic resonance venography (CMRV) has been successfully applied to the central veins [8, 9], it is typically flow-dependent, relatively slow, has thick slices with limited spatial resolution and is sensitive to motion artifact [3, 10, 11]. Therefore, non-contrast CMRV has been used sparingly since the advent of contrast enhanced methods.

Contrast enhanced 3-dimensional (3D) CMRV has been used successfully with both blood pool and extracellular gadolinium based contrast agents (GBCA) [12]. However, in patients with kidney disease, concerns about the risk of nephrogenic systemic fibrosis (NSF) [13–16] has curbed enthusiasm for GBCA enhanced studies, a trend exacerbated by+ recent reports of gadolinium deposition in brain and bone [17, 18].

At the same time, the number of patients requiring dialysis continues to rise [19]. Therefore, a practical and non-nephrotoxic technique for high resolution imaging of the central veins would address a growing unmet clinical need [20–22].

Ferumoxytol is an ultrasmall, superparamagnetic iron oxide (USPIO) nanoparticle, marketed in the U.S. as Feraheme (AMAG Pharmaceuticals, Waltham, Massachusetts, USA) that has been United States Food and Drug Administration (FDA) approved since 2009 for the treatment of iron deficiency anemia in patients with chronic kidney disease. Because of concerns for hypersensitivity reactions from bolus administration of ferumoxytol, a black box warning was issued by the FDA in March, 2015, and new guidelines recommended that ferumoxytol should be administered as a slow intravenous infusion over several minutes [23]. In January, 2018, based on satisfactory supplemental safety data provided by the manufacturer, the FDA broadened the approval to include treatment of iron deficiency in patients with normal renal function in whom oral iron is ineffective or poorly tolerated [24]. Originally designed as an intravascular MR contrast agent, ferumoxytol has been described for a variety of MR applications [25–27]. Its high T1 relaxivity (R1 = ~ 15 mM/s at 1.5 T) and long intravascular half life (~ 15 h) [27, 28] are highly desirable attributes for a venographic imaging agent.

The aims of our study, therefore, were to evaluate the diagnostic performance of 3D ferumoxytol-enhanced CMRV (FE-MRV) for diagnosis of central venous occlusion in patients with renal impairment and to assess its clinical impact on patient management.

## Materials and methods

### Study population

**Fig. 1:**
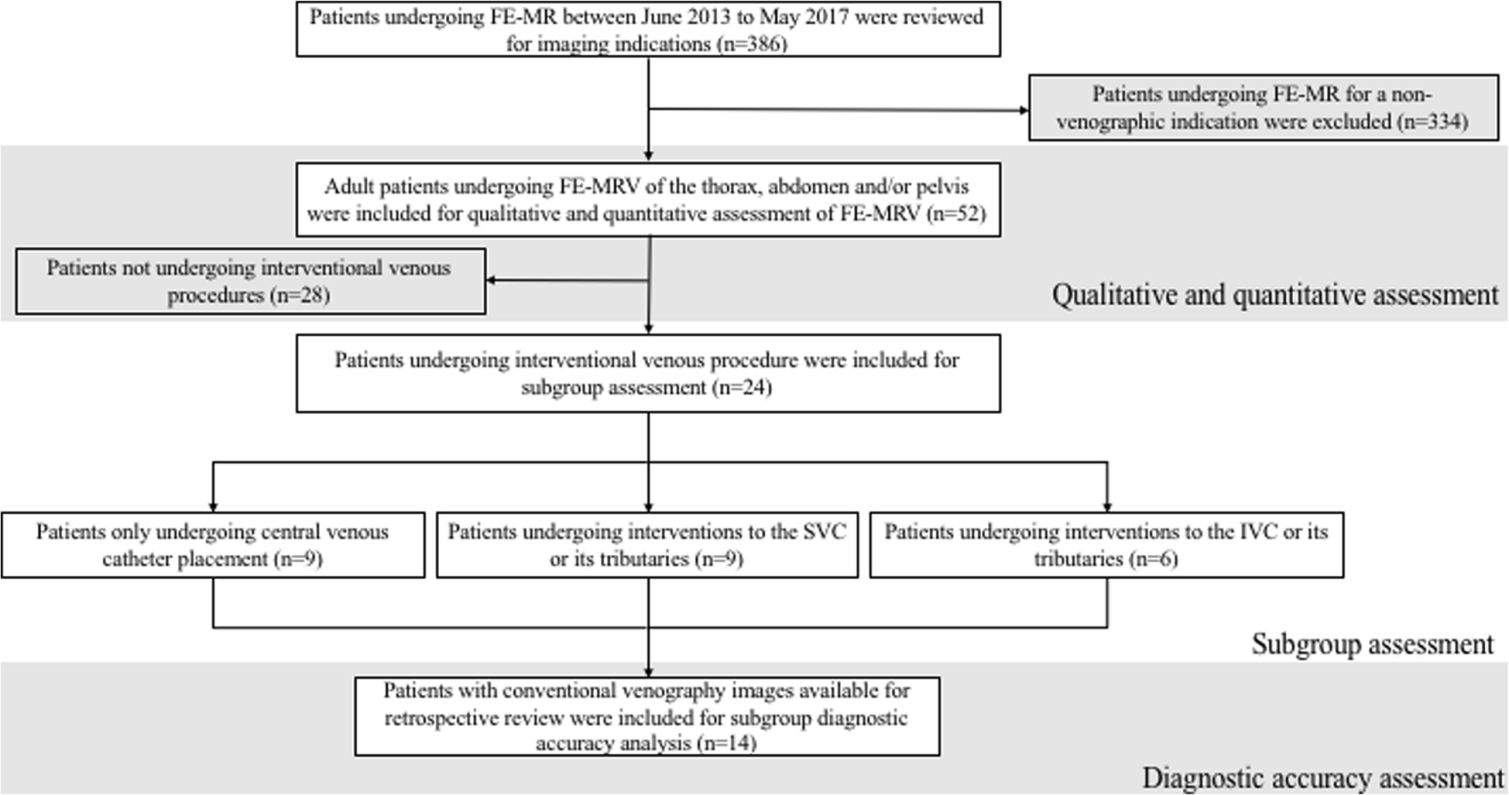
Flowchart demonstrates patient inclusion. Note - The central venous access placement group included only patients that exclusively had this procedure while the superior vena cava (SVC) and inferior vena cava (IVC) reconstruction group included interventions to the respective vessels and/or all their first order tributaries

This retrospective study was approved by the local Institutional Review Board and conformed to the Health Portability and Insurance Accountability Act. All patients provided written informed consent. Fifty-two consecutive adult patients (≥ 18 years) with renal failure (*n* = 48), gadolinium allergy (*n* = 1), insufficient access for fast intravenous infusion of contrast (n = 1) or need for extended-coverage pre-procedural venous mapping (*n* = 2) underwent 3D FE-CMRV of the central veins at a single institution between June 2013 and May 2017. Patient selection criteria are outlined in Fig. 1. Twenty-four patients had interventional venous procedures following the FE-CMRV. Where available, catheter venography (*n* = 14) was used as the reference standard to evaluate the diagnostic accuracy of FE-CMRV.

### Image acquisition

CMR imaging was performed on a 3 T whole body CMR imaging system (Magnetom TIM Trio (*n* = 34), Magnetom Prisma Fit (*n* = 8) or Magnetom Skyra (*n* = 5); Siemens Healthineers, Erlangen, Germany) or on a 1.5 T whole body CMR imaging system (Magnetom TIM Avanto (n = 5), Siemens Healthineers). All patients underwent continuous monitoring of heart rate, blood pressure and pulse oximetry throughout the examination. Stock ferumoxytol (4 mg/kg dose) was diluted ≥6x with normal saline prior to infusion. Two-station, 3D breath-held CMRV of the thorax, abdomen and pelvis was performed during the steady state distribution of ferumoxytol, at least five minutes after infusion. Following the acquisition of the proximal station images, the table position was incremented by 150–200 mm and the second breath held acquisition was performed with the same imaging parameters as the first. Overlapping 3D datasets with identical spatial resolution were then composed inline on the CMR scanning console using proprietary commercial software (Siemens Image Compose). Typical acquisition times were 17–21 s per station with voxel dimensions of 1.0 × 1.2 × 1.3 mm. Prior to April 2015, ferumoxytol was administered intravenously as a bolus over 15 s (*n* = 23); from April 2015 ferumoxytol was administered intravenously by slow-infusion at 0.5 mg/kg/min (*n* = 29), in compliance with FDA guidelines [23]. In all patients, the venographic evaluation was based on the steady state images acquired at least 5 min after infusion, at which time the ferumoxytol distribution is independent of the initial mode of administration (bolus or slow infusion).

Catheter venography was carried out via common femoral vein, internal jugular vein or peripheral vein puncture as required. All procedures were performed by an experienced interventional radiologist or vascular surgeon.

### Qualitative image analysis

Two imaging physicians with 5 and 2 years of experience (B.B. and S.N.K. respectively) in CMR independently evaluated anonymized source and maximum intensity projection (MIP) reconstructed FE-CMRV images (MacOsiriX, Pixmeo, Geneva, Switzerland) for overall image quality, venous segment image quality, presence of collaterals and venous occlusion. Reviewers had full access to all source and MIP images, in addition to Volume Rendered (VR) reconstructions (Vitrea, Vital Images).

The central veins were divided into 21 segments by each reviewer: paired internal jugular, subclavian, brachiocephalic, common iliac, external iliac and internal iliac veins (*n* = 12); superior vena cava (SVC), superior pulmonary veins, inferior pulmonary veins, inferior vena cava (IVC), azygos vein, portal vein, superior mesenteric vein, inferior mesenteric vein, major hepatic veins (*n* = 9). Vascular image quality was scored on a 4-point scale (1 = Vessels not assessable due to poor image quality; 2 = Vessels visualized but only gross features (size/patency) confidently assessable; 3 = Vessels well defined and evaluable for structural pathology with high confidence; 4 = Excellent vessel definition with sharp borders such that fine details can be evaluated with high confidence). Each of the 21 venous segments was assessed for patency or occlusion. Disagreements concerning venous patency were resolved by consensus with a third senior reviewer with 20 years of vascular CMR experience (J.P.F.).

Diagnostic accuracy was determined on retrospectively collected data where venous patency on FE-CMRV was used as the index test and the reference was catheter venography. One board-certified interventional radiologist with 10 years experience in interventional radiology (J.M.M.), blinded to all clinical data, reviewed anonymized fluoroscopic images using a commercial image viewing platform (MacOsiriX, Pixmeo, Geneva, Switzerland). Any discrepancies between the index and reference cases were reviewed blindly by the senior reviewer (J.P.F.). Only vessels imaged on FE-CMRV and catheter venography were included in the diagnostic accuracy assessment, which totaled 92 segments in 14 patients.

### Quantitative image analysis

Signal-to-noise (SNR) and contrast-to-noise (CNR) ratios of the IVC relative to the aorta were measured by a single reviewer (P.S.) after drawing circular regions of interest (ROIs) over the IVC, adjacent abdominal aorta, and adjacent hepatic tissue. Three background ROIs were drawn outside of the image. The ROIs were drawn to maximize coverage area but avoided contamination by obvious image artifacts such as those produced from IVC filters. Noise was defined as the average of the standard deviation of the background signal intensity. The SNR was calculated by dividing the signal intensity of the IVC or aorta by the noise; the CNR was calculated by dividing the difference in signal intensity between the IVC or aorta and adjacent tissue by the noise. No additional normalization or isolation procedures for the effect of parallel acquisition were employed, because the IVC and aorta were subject to the same additional noise and the acquisition parameters represented those typically used for clinical imaging.

### Measures of clinical outcome

As a measure of added value for FE-CMRV in clinical practice, the influence of FE-CMRV on clinical outcomes and patient management was assessed. A review of the electronic medical record was conducted to determine the requirement for additional imaging, changes in renal function, serum-iron levels, interventional parameters, and adverse event (AE) rate. The contrast and radiation doses for the interventional procedures were categorized in three subgroups depending on the type of intervention (Fig. 1). For renal function, serum creatinine and estimated glomerular filtration rate (eGFR) prior to the FE-CMRV and prior to and following the interventional procedures were collected. Following catheter intervention, renal function was evaluated up to 48 h and the earliest laboratory values following the intervention were recorded. Serum-iron values before and after ferumoxytol administration were noted if measured within one month of the injection. Additional imaging was defined as imaging carried out for the same indication in the interim between the FE-CMRV and intervention or final clinical management decision.

### Statistical analysis

Continuous data are presented as means and standard deviation (SD) or medians and interquartile range (IQR). Categorical data are presented as absolute values and relative frequencies (percentages). Data were tested for normality using the Shapiro-Wilk test. Group differences were compared using paired two-tailed t-tests or Wilcoxon rank-sum test as appropriate. Interobserver agreement was determined using Gwet’s AC1 statistic because of “kappa’s paradox” [29]. The agreement by AC1 was assessed as: 0.00–0.20, poor; 0.21–0.40, fair; 0.41–0.60, moderate; 0.61–0.80, good; 0.81–1.00, very good. Sensitivity, specificity, positive and negative predictive values, and accuracy were determined for FE-CMRV. Statistical analysis was performed by using SPSS (version 25.0; SPSS, Chicago; Illinois, USA). Differences with a *P* value of less than 0.05 were considered statistically significant.

## Results

### Patient characterisitics

Table 1 summarizes demographic data for all 52 patients undergoing FE-CMRV. The median age was 47 years (IQR 32–61) and 23 (44%) were female. Forty-four (85%) of the patients had chronic kidney disease (CKD), four (8%) had acute kidney injury (AKI) and four (8%) had no renal impairment at baseline. Forty-two (81%) patients had Stage 3 CKD or above and 23 (44%) patients were on dialysis. In the 29 (56%) patients not on dialysis, the mean pre-FE-CMRV creatinine and eGFR was 2.2 ± 1.1 mg/dL and 41.7 ± 25.6 mL/min/1.73 m^2^ respectively. The primary indication for FE-CMRV was suspected venous occlusion (33 of 52 patients, 63%) or venous road mapping prior to central venous catheter placement (19 of 52 patients, 37%). Twenty-four patients (46%) underwent venous intervention following pre-procedural FE-CMRV road mapping. The median time from FE-CMRV to intervention was 3.5 days (IQR 2.0–7.0). No AE occurred following FE-CMRV or catheter venography. Vital signs remained stable throughout the course of the FE-CMRV and catheter venography exams. In 3 patients in whom serum iron levels were measured before ferumoxytol administration and at follow up, the levels did not change significantly from baseline (*p* = 0.65).

**Table 1:** Patient characteristics

	All patients (*n* = 52)	Interventional procedure (*n* = 24)	No interventional procedure (*n* = 28)
Female sex^a^	23 (44)	10 (42)	13 (46)
Age (y)^b^	47 (32–61)	47 (28–63)	44 (32–59)
Chronic kidney disease^a^	44 (85)	21 (88)	23 (82)
Hemodialysis	22 (42)	10 (42)	12 (43)
Peritoneal dialysis	1 (2)	1 (4)	0
No dialysis	29 (56)	13 (54)	16 (57)
Transplant	13 (25)	7 (29)	6 (21)
Chronic kidney disease stage^a^			
Stage 5	25 (48)	13 (54)	12 (43)
Stage 4	9 (17)	3 (13)	6 (21)
Stage 3	8 (15)	5 (21)	3 (11)
Stage 2	1 (2)	0	1 (4)
Stage 1	1 (2)	0	1 (4)
Acute kidney injury^a^	4 (8)	2 (8)	2 (7)
Comorbidities^a^			
Diabetes mellitus	17 (33)	6 (25)	11 (40)
Diabetic nephropathy	5 (10)	3 (13)	2 (7)
Hypertension	33 (63)	14 (58)	19 (68)
Heart failure	6 (12)	3 (13)	3 (11)

^a^Data are number of patients, with percentages in parenthesis. Percentages were rounded

^b^Data are median, with interquartile range in parenthesis

### FE-CMRV qualitative image analysis

The overall image quality scores for the 52 FE-CMRV examinations was 3.92 ± 0.27 (observer A) and 3.81 ± 0.40 (observer B), with good interobserver agreement (AC1 = 0.79; 95% CI: 0.67, 0.91). In all cases, the overall image quality score was ≥3, such that vessels could be evaluated for structural pathology with high confidence. 1033 of 1038 (99.5%) of venous segments were considered of diagnostic quality (score ≥ 2) with very good interobserver agreement (AC1 = 0.91; 95% CI: 0.89, 0.92). The interobserver agreement for the presence of venous occlusion was very good (AC1 = 0.93; 95% CI: 0.92, 0.95); disagreement in 5% (53/1033) of the venous segments was resolved by consensus. The interobserver agreement for the presence of collaterals in patients with central venous occlusion (*n* = 36) was moderate (AC1 = 0.46; 95% CI: 0.22, 0.70).

**Fig. 2:**
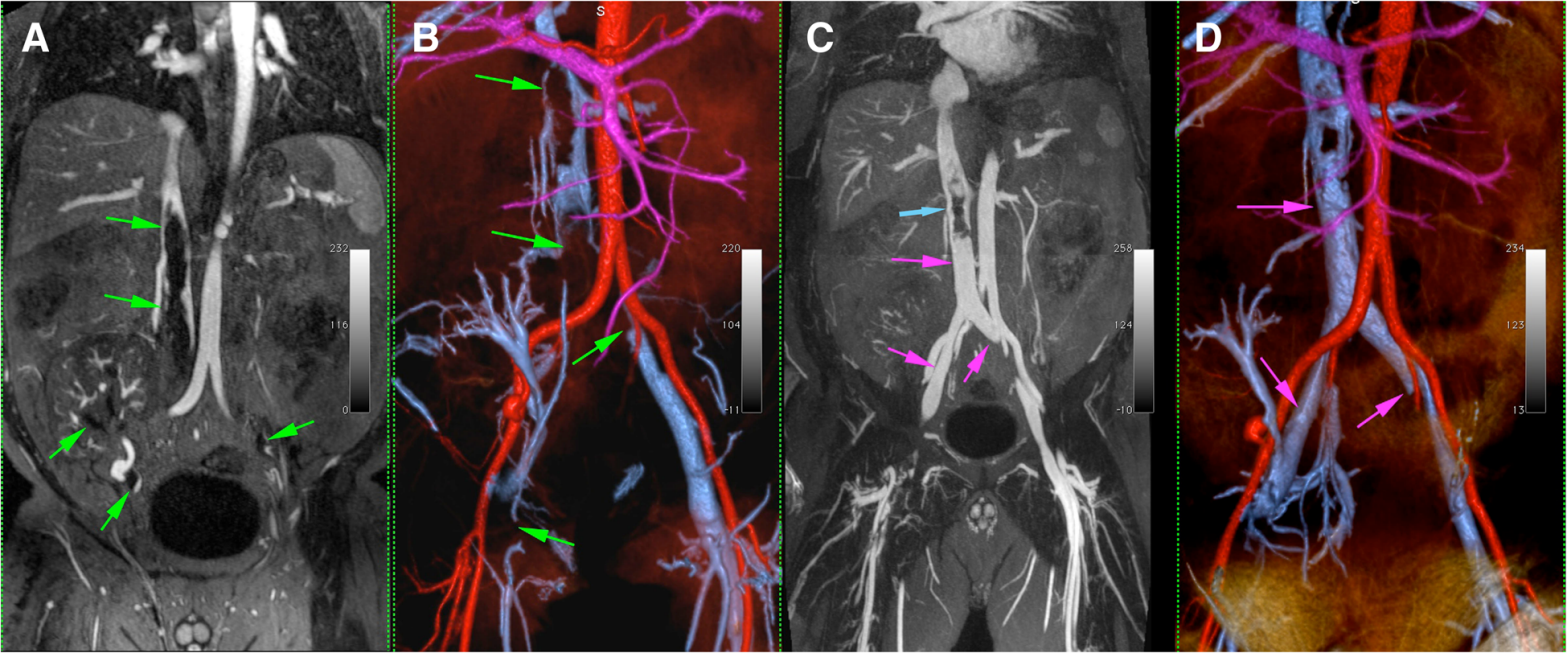
47 year-old male with end-stage renal disease post-transplantation presented with persistent right lower leg swelling 8 days after IVC filter placement. Initial 1.5 T FE-CMRV source image (**a**) and color 3D volume rendering (**b**) show extensive occlusion extending from the inferior vena cava (IVC) and bilateral common iliac veins to the right renal transplant vein and right common femoral vein (green arrows in **a** and **b**). Following intervention (**c** and **d**), the IVC and common iliac veins are largely recanalized (purple arrows in **c** and **d**) and the IVC filter (blue arrow in **d**) is in good position

**Fig. 3:**
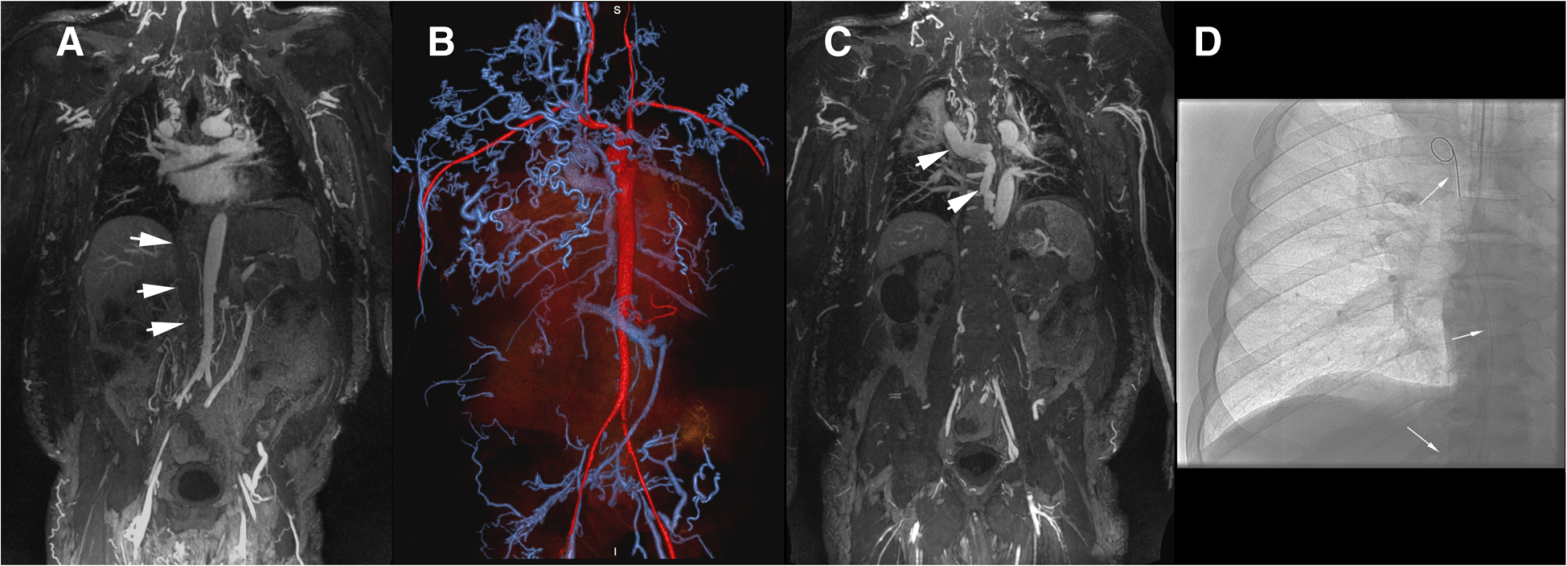
56 year-old male with end-stage renal disease presented with loss of hemodialysis access and multiple failed attempts at central venous access. 3 T FE-CMRV source images (**a** and **c**) and reconstructed color 3D volume rendering (**b**) demonstrate complete occlusion of the infrahepatic inferior vena cava (white arrows in A) and dilated azygos vein (white arrows in **c**) with extensive collateralization. Fluoroscopic image (**d**) demonstrates percutaneous transhepatic inferior snare technique through a patent hepatic vein (white arrows in **d**) for successful Permacatheter tip placement in the right atrium performed based on the vascular map provided by FE-CMRV and without iodine based contrast media

**Fig. 4:**
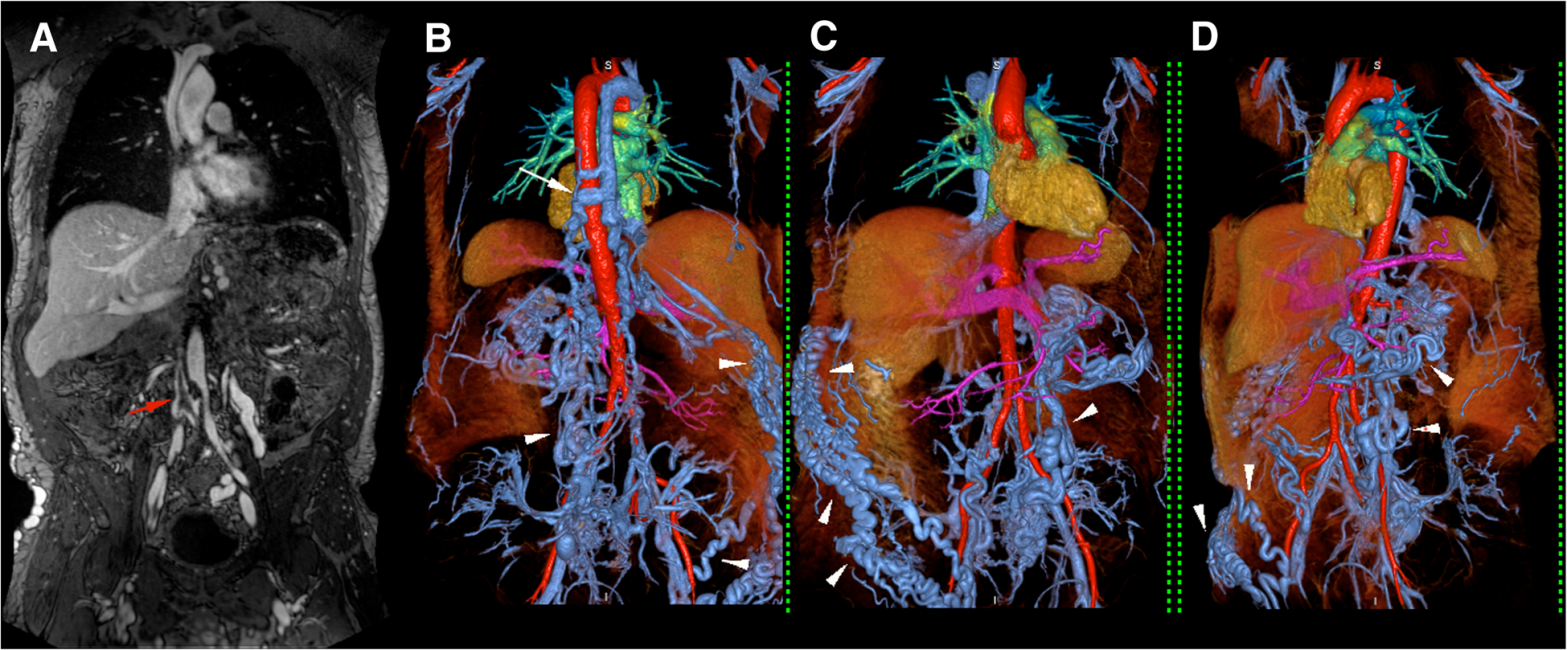
Pre-procedural venous mapping in a 55 year old male patient. 3 T FE-CMRV source image (**a**) and color 3D volume rendering (**b**-**d**) show thrombosis at the confluence of the common iliac veins (red arrow in **a**) with extensive collaterals (white arrowheads in **b**-**d**) and an enlarged azygos vein (white arrow in **b**) draining to the superior vena cava

**Fig. 5:**
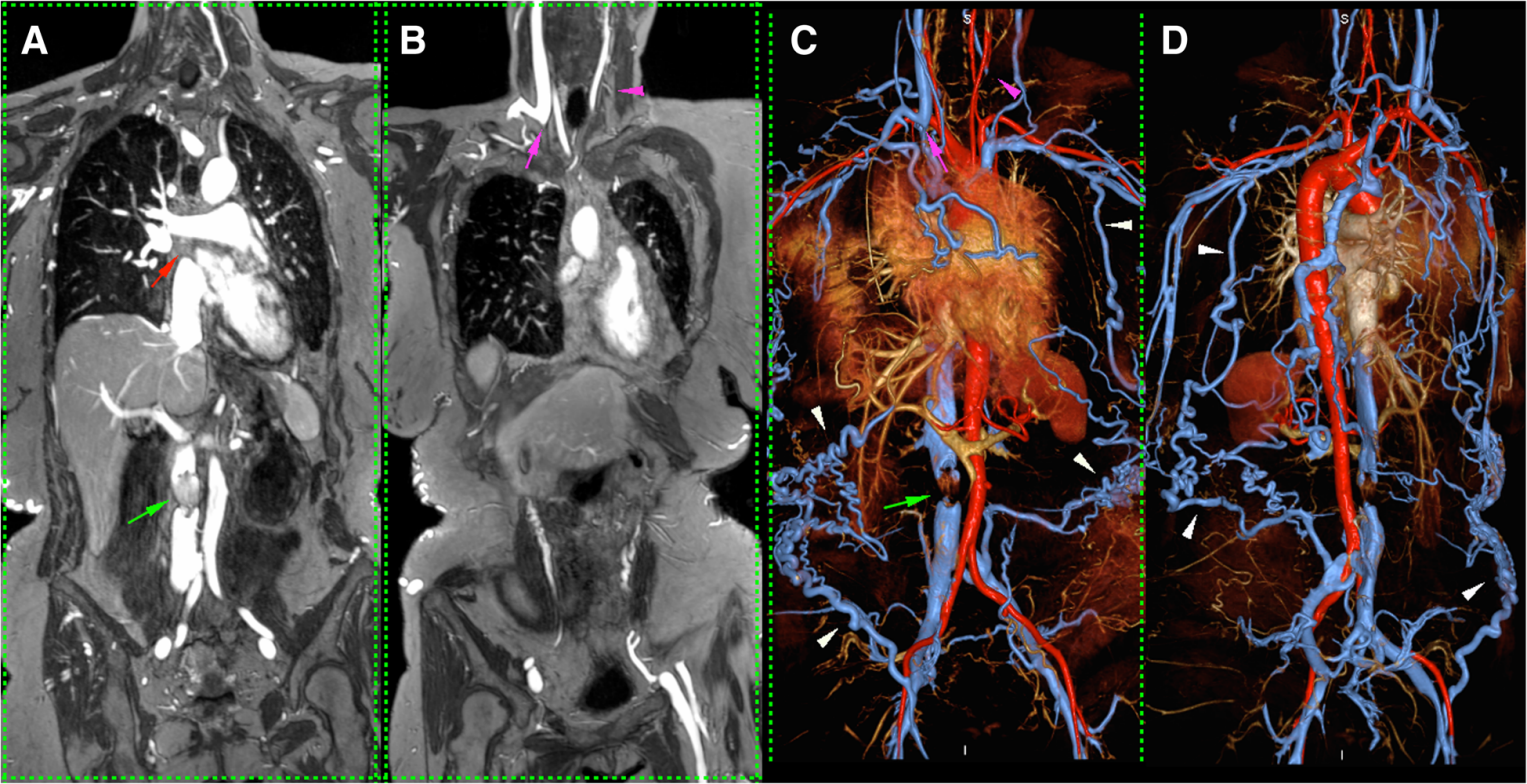
49 year-old female with Stage 4 chronic kidney disease presented with worsening varicosities. 3.0T FE-CMRV maximum intensity projection (**a**, **b**) and color 3D volume rendering (**c**, **d**) show patent inferior vena cava (IVC) with IVC filter in place (green arrow in **a** and **c**) and occlusion of the superior vena cava (red arrow in A), right brachiocephalic vein (purple arrow in **b** and **c**) and left internal jugular vein (purple arrowhead in **b** and **c**). Extensive collaterals to the pelvic veins are clearly visible (white arrowheads in **c** and **d**)

**Fig. 6:**
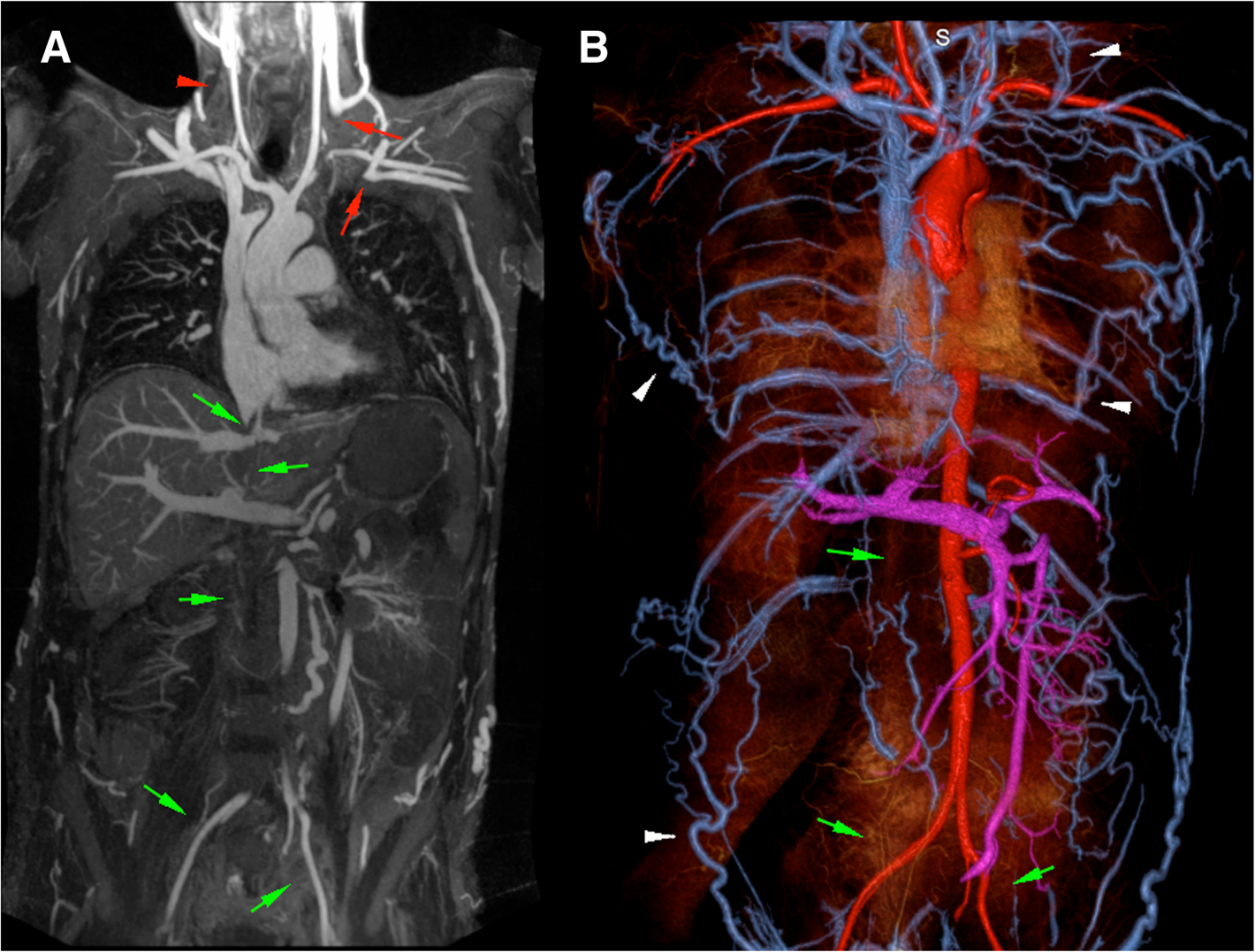
33 year-old male with end-stage renal disease on hemodialysis required venous mapping prior to central venous access. 1.5 T FE-CMRV maximum intensity projection (**a**) and color 3D volume rendering (**b**) show occluded right internal jugular and subclavian veins (red arrows in **a**), non-visualized occluded right internal jugular vein (red arrowhead in **a**) and complete occlusion of the entire inferior vena cava and common iliac veins (green arrows in **a** and **b**). Collateral veins are highlighted in **b** (white arrowheads)

Figures 2, 3, 4, 5, and 6 illustrate representative FE-CMRV studies with MIP and VR reconstructions. The extended field of view (FOV) coverage is evident as is the consistent visualization of occlusions and collateralization. Figure 2 illustrates partial resolution of extensive venous occlusion following thrombectomy and thrombolysis; Fig. 3 illustrates multiple occlusions and collateralization and this study facilitated placement of a central venous catheter without iodine based contrast media; Fig s. 4, 5, and 6 illustrate central venous occlusion and widespread collateralization. Additional file 1 summarizes the venous segment image quality, where segments with lateralization were grouped together.

### FE-CMRV quantitative image analysis

There was no significant difference between the SNR or CNR of the IVC and adjacent aorta (*p* = 0.76 for both) on FE-CMRV images.

The sensitivity, specificity, positive and negative predictive value and accuracy of FE-CMRV was 92.6, 96.9, 92.6, 96.9 and 95.7%. After discrepancies between the FE-CMRV index cases and catheter venography were resolved by the senior reviewer, the overall sensitivity, specificity, positive and negative predictive value and accuracy was 100.0% for all (Table 2).

**Table 2:** Diagnostic Accuracy of Ferumoxytol Enhanced Cardiovascular Magnetic Resonance Venography (FE-CMRV)

Reader	No. of TP Findings	No. of TN Findings	No. of FP Findings	No. of FN Findings	Sensitivity	Specificity	PPV	NPV	Accuracy
Consensus^a^	25	63	2	2	92.6 (75.7–99.1)	96.9 (89.3–99.6)	92.6 (89.2–99.2)	96.9 (89.2–99.2)	95.7 (89.2–98.8)
Ultimate assessment^b^	27	65	0	0	100.0 (87.2–100.0)	100.0 (94.5–100.0)	100.0	100.0	100.0 (96.1–100.0)

^a^Consensus read was determined by consensus of the primary reviewers with a third senior reviewer in cases where there was disagremment between the reviewers A and B for the FE-CMRV

^b^Ultimate assessment was determined by the senior reviewer (J.P.F.) for cases with disagreement between the consensus FE-CMRV and reference standard

Note – Data in parenthesis are 95% CIs. FN = false-negative, FP = false-positive, NPV = negative predictive value, PPV = positive predictive value, TN = true-negative, TP = true-positive

**Fig. 7:**
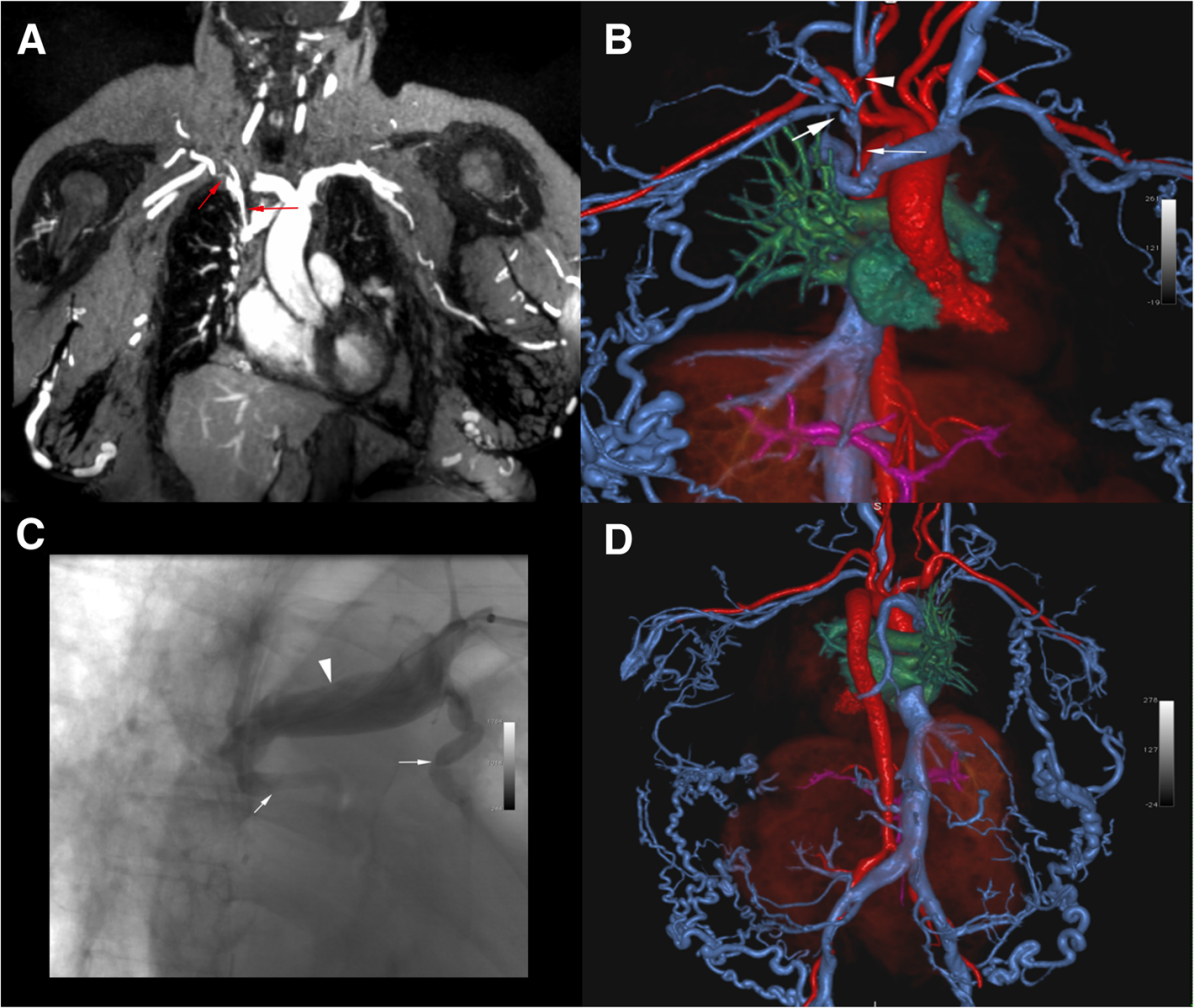
Example of a false-positive score of the subclavian vein in a 67 year-old female with end-stage renal disease and upper extremity swelling. 3 T maximum intensity projection FE-CMRV (**a**) and color 3D volume rendering (**b**) show two foci of high-grade stenosis of the right subclavian vein (red arrows in A and white arrows in **b**) and right internal jugular occlusion (white arrowhead in **b**), initially interpreted as occluded. Catheter venography (**c**) confirms subclavian vein stenoses (white arrows in **c**) and dilated proximal subclavian vein (white arrowhead in **c**). Full field-of-view color 3D volume rendering (**d**) shows extensive venous collaterals throughout the body

Representative examples of initial results that were reclassified by the senior reviewer are provided in Fig. 7 and Additional file 2. Figure 7 highlights the difficulty in distinguishing high-grade stenoses from complete occlusions in the setting of proximal dilation and extensive collateralization leading to a false-positive result. Additional file 2 is an example of a false-negative result where an occluded venous segment was initially interpreted as patent. The average time interval between the FE-CMRV and catheter venogram was 14.0 days (range 0 to 98 days).

### Clinical outcomes

Table 3 summarizes the procedure-related outcomes following venous intervention, including the requirement for follow-up imaging, contrast and radiation doses and renal function status. Of note, no patient required additional imaging following FE-CMRV and prior to intervention.

**Table 3:** Clinical outcomes in patients undergoing post-CMR intervention^a^

Parameter	All patients (*n* = 24)	Only central venous access placement (*n* = 9)	SVC reconstruction (*n* = 9)	IVC reconstruction (*n* = 6)
Pre-intervention Creatinine^a^	4.0 (3.5)	4.6 (4.0)	4.6 (4.0)	2.3 (1.4)
Post-intervention Creatinine^a^	3.6 (2.9)	4.1 (3.7)	4.0 (2.8)	2.1 (1.2)
Pre-intervention eGFR^a^	30.9 (26.0)	31.8 (33.5)	21.8 (13.7)	43.2 (25.9)
Post-intervention eGFR^a^	31.0 (23.5)	31.4 (28.0)	21.4 (12.4)	44.7 (25.6)
Days from MRI to interventional procedure^b^	3.5 (2.0–7.0)	2.0 (0.5–4.0)	7.0 (2.5–28.5)	3.0 (1.5–16.3)
Received iodine based contrast media^c^	15 (63)	1 (11)	9 (100)	5 (83)
Volume of iodine based contrast media (mL)^b^	12.5 (0–40.0)	0 (0–0)	30.0 (17.5–60.0)	42.5 (7.5–52.5)
Fluoroscopy time (minutes)^b^	10.0 (1.9–25.1)	1.4 (0.3–2.4)	16.0 (7.3–29.0)	23.1 (10.1–49.0)
Air Kerma (mGy)^b^	341.0 (20.0–1337.0)^d^	15.0 (9.5–29.7)	450.5 (346.8–1476.5)^d^	1479.4 (610.8–2283.0)
Dose area product (cGy/cm^2^)^b^	4590.2 (423.1–20,302.1)^d^	432.1 (238.3–858.9)	9806.8 (5736.7–18,852.4)^d^	44,273.8 (3485.1–59,494.6)

^a^Data are mean, with standard deviation in parenthesis

^b^Data are median, with interquartile range in parenthesis

^c^Data are number of patients, with percentages in parenthesis. Percentages were rounded

^d^The air kerma and dose area product could not be extracted from the chart of one patient undergoing an SVC reconstruction procedure

Procedural contrast was not used during catheter intervention in nine (38%) out of 24 patients: eight patients had central venous catheters placed and one patient underwent intravascular ultrasound-guided placement of a thrombolysis catheter in the IVC.

The interventional radiation exposure for all procedures was reflected by the fluouroscopy time, air kerma and dose area product (DAP) which were 10.0 min (IQR 1.9–25.1), 341.0 mGy (IQR 20.0–1337.0) and 4590.2 cGy/cm^2^ (IQR 423.1–20,302.1) respectively. The radiation doses associated with central venous placement were 1.4 min (IQR 0.3–4.0), 15.0 mGy (IQR 9.5–29.7) and 432.1 cGy/cm^2^ (IQR 238.3–859.9) respectively.

Twenty-eight (54%) of 52 patients did not undergo an intervention following the FE-CMRV. Central venous occlusion was confidently excluded in eleven (39%) out of 28 patients who were managed conservatively. Follow up over a month did not show any evidence of central venous occlusion in these patients. In the remaining 17 (61%) patients not undergoing intervention, central venous occlusion was identified on FE-CMRV, but these patients were conservatively managed because of clinical non-progression or anticipated low benefit-to-risk ratio of intervention (*n* = 15); patient preferences (n = 1); or ultrasound-guided central catheter placement by the liver transplant team (n = 1). No additional imaging was needed in any of the 28 patients not undergoing intervention to make a conservative management decision. Thus, no patient had additional imaging to guide intervention or conservative management in our study.

## Discussion

The results of our study suggest that 3D FE-CMRV is a highly accurate and reliable technique for imaging central venous anatomy in patients with CKD and can be used to inform image-guided therapy. FE-CMRV adds value by providing comprehensive assessment of venous anatomy without the need for additional diagnostic imaging. FE-CMRV facilitated venous intervention with short latency between diagnosis and treatment, and likely minimized the overall requirement for intra-procedural iodine based contrast media. Moreover, the ability of FE-CMRV confidently to exclude venous disease when absent informed conservative clinical management. Streamlining imaging and intervention is particularly important in renally impaired patients who have multiple comorbidities and are at increased risk of adverse events and procedural complications.

The stable, steady state vascular signal that is characteristic of ferumoxytol eliminates the time dependency between infusion and imaging. Once ferumoxytol is distributed in the vascular space, even vessels that are slow to fill or have high capacitance become enhanced to the same degree as the fastest filling vessels. This is a powerful attribute in that failure to enhance is diagnostic of occlusion. The same mechanism makes it possible to visualize venous collaterals with striking clarity in a non-time-dependent fashion, such that FE-CMRV promises to set a new standard for high resolution central venous imaging.

Although no agreed upon reference standards exist for radiation doses in the interventional procedures carried out in our study, the central tendencies and ranges are on the same order as other workers have reported, including the Radiation Doses in Interventional Radiology (RAD-IR) study [30, 31]. Some patients in our study had highly complex intervention, such that one might expect higher radiation doses than in less complex procedures [32]. Our patient with the longest fluoroscopy time had a very high thrombotic burden requiring combined antegrade and retrograde recanalization of the IVC via popliteal and internal jugular vein access. In this patient, the procedure comprised Angiovac thrombectomy, balloon venoplasty of the IVC and bilateral iliac veins and mechanical thrombectomy. There is a scarcity of reports regarding contrast volumes used for common venous procedures in the literature, but it is generally considered that contrast volumes < 100 mL are preferred to avoid contrast-induced nephropathy (CIN) [33]. Of note, none of the procedures in our study required contrast volumes higher than 100 mL, and 8/9 (89%) catheter placement procedures required no contrast media. These findings suggest that FE-CMRV may help minimize exposure to iodine based contrast media and radiation not only for pre-procedural imaging, but also during intervention.

Ferumoxytol is known to have high r1 relaxitivity and a long intravascular half-life [28], which supports unhurried, extended field-of-view imaging. Our study found no difference in the steady state SNR or CNR between the IVC and aorta, consistent with the stable intravascular distribution of ferumoxytol.

Because ferumoxytol eliminates the risk of contrast-induced nephropathy, NSF and concerns about gadolinium deposition, it an attractive alternative to CT venography and gadolinium enhanced CMRV in patients with kidney disease. As a secondary effect, patients may derive therapeutic benefit from ferumoxytol administration, since the majority of patients with CKD have iron deficiency anemia. This is a unique attribute for a diagnostic imaging contrast agent.

At the same time, vigilence must be exercised to monitor for unanticipated hypersensitivity reactions. In clinical trials of therapeutic use where ferumoxytol was administed as a tight (30 mg /sec) bolus, the serious AE rate was 0.2% [34]. Ferumoxytol was used therapeutically in this way from 2009 to 2015. In March, 2015, based on post marketing reports, the FDA issued a black box warning about potential hypersensitivity reactions and withdrew approval for bolus administration [23]. Updated FDA guidelines as specified in the package insert now require slow intravenous infusion, similar to the other intravenous iron therapy agents. The FDA recognizes that post-marketing AE data are more difficult to interpret than those in clinical trials and it is generally more difficult to infer a cause and effect relationship between the agent and the event. Moreover, in January, 2018, the FDA expanded the approval for ferumoxytol therapy to include patients without renal impairment who are intolerant of oral iron or in whom oral iron is ineffective [24].

For diagnostic use, several single-center studies of ferumoxytol-enhanced CMR have shown no serious AE and very few minor AEs [35–38]. Nonetheless, we have updated our practice guidelines in compliance with FDA recommendations. We infuse ferumoxytol slowly and we monitor patients closely, during and for at least 30 min after administration. Further safety data will be required before the true incidence of AE associated with the diagnostic use of ferumoxytol is fully defined. To this end, a multi-center ferumoxytol Registry has been established [39] to support evidence-based practice guidelines for the safe and appropriate use of the agent in a broader clinical context.

Several limitations of our study warrant discussion. First, the number of vessels used for the diagnostic accuracy assessment was relatively low. The limiting factor was the number of catheter images because these were available only for vessels that were injected and relevant to the clinical procedure. Nonetheless, the analysis spanned the majority of venous segments and thus decreases the risk of a potential selection bias. The long interval between the FE-CMRV and some catheter studies (up to 98 days) can cause a length time bias. Despite this, the agreement between both modalities was very high. FE-CMRV of the central veins has already shown promising results in small pediatric cohorts [40–42] and in patiens with pelvic vein thrombosis [43], but our study addresses a large adult cohort and establishes diagnostic accuracy and value added to patient care and management.

## Conclusion

3D FE-CMRV is a practical, accurate and robust technique for mapping the central thoracic and abdominal veins. FE-CMRV promises to set a new standard for non-invasive, high resolution venous imaging, informs image-guided intervention and provides a viable option to patients in whom conventional contrast agents may be contraindicated or ineffective.

## Additional files

Additional file 1: Vessel segment image quality by observer A and B of 15 venous segments. Image quality was assessed on a 4-point scale (1 = Vessels not assessable due to poor image quality; 2 = Vessels visualized but only gross features (size/patency) confidently assessable; 3 = Vessels well defined and evaluable for structural pathology with high confidence; 4 = Excellent vessel definition with sharp borders such that fine details can be evaluated with high confidence). Data are number of segments with percentages in parenthesis. Percentages were rounded. *Right and left vessels were grouped together for the overall observer score. Note - SVC = superior vena cava, BCV = brachicephalic vein, IJV = internal jugular vein, SUB = subclavian vein, SUP-P = superior pulmonary vein, INF-P = inferior pulmonary vein, AZY = azygos vein, SMV = superior mesenteric vein, IMV = inferior mesenteric vein, MHV = main hepatic vein, IVC = inferior vena cava, PV = portal vein, CIV = common iliac vein, IIV = internal iliac vein, EIV = external iliac vein. (TIF 2516 kb)

Additional file 2: Example of a false-negative score of the subclavian vein in a 20 year-old female with end-stage renal disease. 3 T FE-CMRV source image (A) and color 3D volume rendering (B) show complete occlusion the right subclavian vein (white arrows in A and B), initially interpreted as severely narrowed. Catheter venography (C-D) confirms the subclavian vein occlusion (white arrow in D). (TIF 24500 kb)

## Abbreviations

AE: Adverse event
AKI: Acute kidney injury
CI: Confidence interval
CIN: Contrast induced nephropathy
CKD: Chronic kidney disease
CMR: Cardiovascular magnetic resonance
CMRV: Cardiovascular magnetic resonance venography
CNR: Contrast-to-noise ratio
CT: Computed tomography
eGFR: Estimated glomerular filtration rate
FDA: Food and Drug Administration
FE: Ferumoxytol-enhanced
FOV: Field of view
GBCA: Gadolinium-based contrast agent
IQR: Interquartile range
IVC: Inferior vena cava
MIP: Maximum image projection
MRV: Magnetic resonance venography
NSF: Nephrogenic systemic fibrosis
ROI: Region of interest
SD: Standard deviation
SNR: Signal-to-noise ratio
SVC: Superior vena cava
VR: Volume rendered
