# Supplementary figures and images for: High resolution, 3-dimensional Ferumoxytol-enhanced cardiovascular magnetic resonance venography in central venous occlusion
Source: J Cardiovasc Magn Reson. 2019 Mar 11;21:17. doi: 10.1186/s12968-019-0528-5 (PMC6410526; doi:10.1186/s12968-019-0528-5)

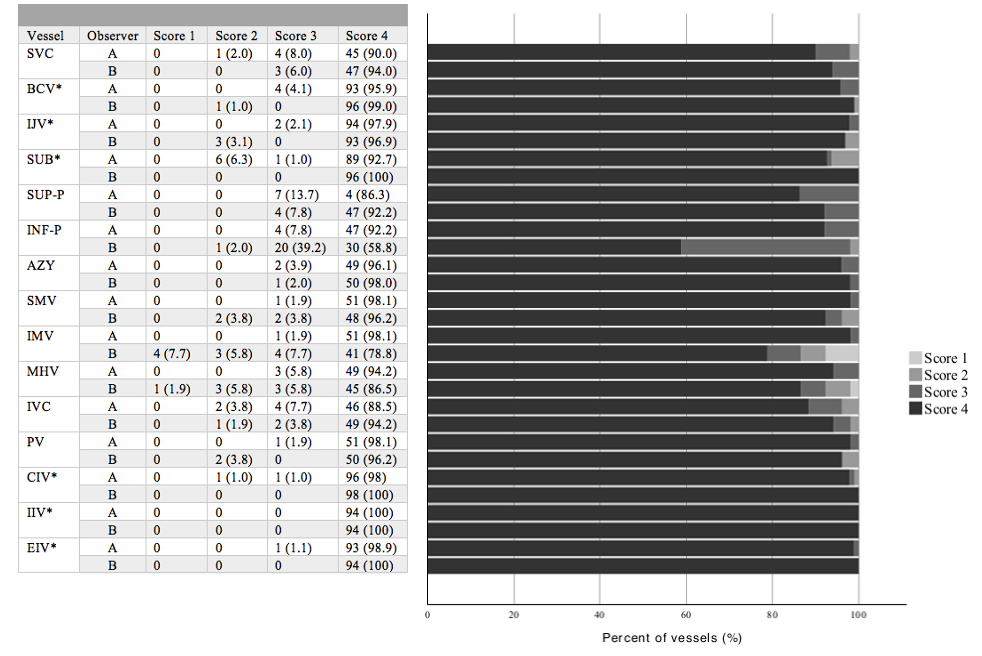

Supplement: Supplementary file 1 — Vessel segment image quality by observer A and B of 15 venous segments. Image quality was assessed on a 4-point scale (1 = Vessels not assessable due to poor image quality; 2 = Vessels visualized but only gross features (size/patency) confidently assessable; 3 = Vessels well defined and evaluable for structural pathology with high confidence; 4 = Excellent vessel definition with sharp borders such that fine details can be evaluated with high confidence). Data are number of segments with percentages in parenthesis. Percentages were rounded. *Right and left vessels were grouped together for the overall observer score. Note - SVC = superior vena cava, BCV = brachicephalic vein, IJV = internal jugular vein, SUB = subclavian vein, SUP-P = superior pulmonary vein, INF-P = inferior pulmonary vein, AZY = azygos vein, SMV = superior mesenteric vein, IMV = inferior mesenteric vein, MHV = main hepatic vein, IVC = inferior vena cava, PV = portal vein, CIV = common iliac vein, IIV = internal iliac vein, EIV = external iliac vein. (TIF 2516 kb) [file 12968_2019_528_MOESM1_ESM.tif]

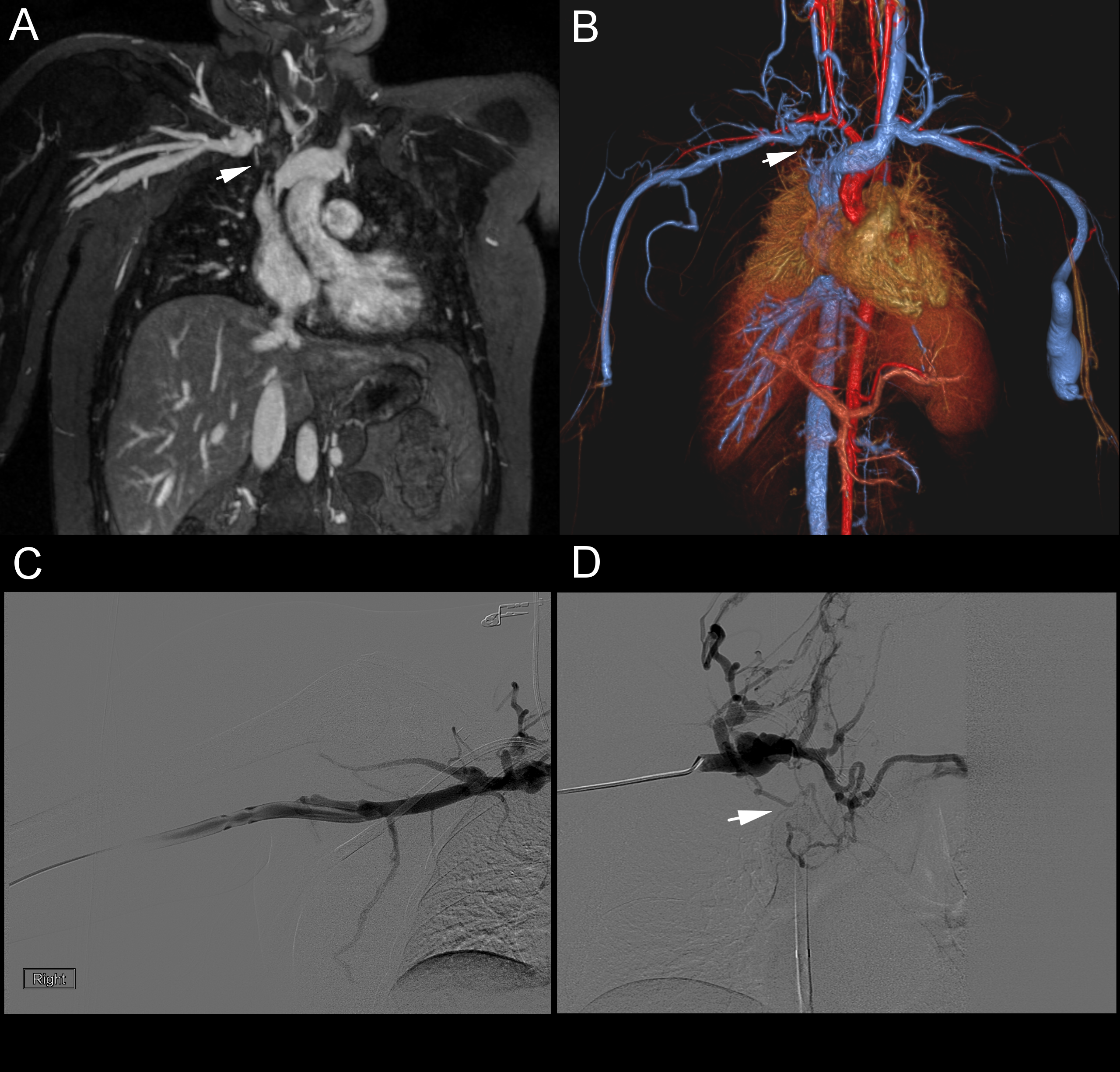

Supplement: Supplementary file 2 — Example of a false-negative score of the subclavian vein in a 20 year-old female with end-stage renal disease. 3 T FE-CMRV source image (A) and color 3D volume rendering (B) show complete occlusion the right subclavian vein (white arrows in A and B), initially interpreted as severely narrowed. Catheter venography (C-D) confirms the subclavian vein occlusion (white arrow in D). (TIF 24500 kb) [file 12968_2019_528_MOESM2_ESM.tif]
